# Some Characteristics of Kali-phosphoricum

**Published:** 1892-04

**Authors:** E. E. Case

**Affiliations:** Hartford, Conn.; Bureau of Materia Medica, I. H. A.


### SOME CHARACTERISTICS OF KALI-PHOSPHORICUM.

### AS EXPERIENCED IN PROVING 30TH AND 100TH (B. & T.), 1M (TYRRELL), AND 40M (F.)

E. E. CASE, M.D., HARTFORD, CONN.

(Bureau of Materia Medica, I. H. A.)

### MIND.

Despondency.

Disinclination to converse with others.

Memory defective for even familiar names.

Difficulty in getting words to express ideas.

In general a sluggish condition of mind, which will act if aroused. A condition much like that experienced after preparing for and passing through an important examination. The physical condition resembles this.

### HEAD.

Dull aching in the occiput extending through the base of the brain, with the peculiar characteristic which has been verified clinically, better by eating. Also relief from eructations of gas. Also better from gentle motion (clinical).

This seems to be a hungry headache.

Pain in the occiput and lumbar region when awaking in the morning; better by lying on the back, passing off after rising. (This occurred on the fourth, and again from the nineteenth to the twenty-first day with the 40M). In many instances during the proving of the 40M, a return of symptoms was noted in a period of from ten to fourteen days.

Sharp, sticking pains outward from the orbits to the temples, better by pressure on the temples.

Intense itching of the scalp, worse at night whenever awake. The higher the potency the more severe, and long continued were all symptoms of the skin.

### Eyes.

Conjunctivæ much affected—as in all stages of catarrhal conjunctivitis, from dryness and redness in the mucous discharges, and agglutinated lids, sensation as from sticks and sand under the lids.

Eyeballs sore, painful when touched or turned.

### Ears.

Itching in the auditory canals.

Pimples about the meatus of left ear.

### Nose.

Sneezing, with bland coryza, worse in the open air.

Thick, yellow mucous discharge, sticky, forming crusts in the nares at night which are offensive to the smell. Bloody crusts in the morning; sometimes epistaxis of dark blood follows their removal.

Sores inside the nares.

Tip of nose cracked.

Blisters on margin of alæ coalescing into one crust.

The catarrhal symptoms show a relationship to Kali-bich., but instead of being ropy, the discharge, though tenacious, is more cohesive than adhesive. This catarrh would persist for at least two weeks in all provings.

### Face.

Intense itching under the beard, also itching pimples, neuralgic stitches, especially from the upper teeth to the ear on left side, and from the temple near the ear to a point above the eye on the right side, while driving, is less in the open air; better by warmth of the hand (nineteenth to the twenty-first day, gradually decreasing with the 40M).

### Mouth.

Thick white coating on the tongue in the morning.

Offensive odor from the mouth.

Foul taste, sometimes bitter.

Saliva profuse, thick, salty.

Gums pale, swollen, easily bleeding.

Roof of mouth swollen, lying in ridges.

A dead tooth (with fistulous opening) ulcerated.

Grumbling pain in the teeth, which were sore in their sockets.

Dryness in the throat, and sensation as if grain husks were in it.

Tonsils swollen, and aching; left most affected.

A point at the right of the larynx, was affected in all the provings in different degrees, lameness, swellings, achings, and from the 30M an abscess which discharged internally. With the 40M on the morning of the twentieth day, both tonsils had upon them solid white deposits, looking like the membranes of diphtheria, traces of the same were upon the pillars of the fauces. The throat became clear toward night. A similar deposit was upon the left tonsil the next morning, but passed away before noon.

Pain from the left tonsil to left ear, worse in open air upon the same days, also fever, prostration and general aching, better by slight motion.

### Stomach.

Appetite increased on the first few days, then decreased, and lost during the remainder of the proving.

Gaseous eructations (after eating) would begin soon after first dose; qualmishness or nausea felt from the stomach to the throat; better by eructation of gas.

Empty gnawing; faint or trembling sensations in the stomach; better temporarily after eating.

### Abdomen.

Distended with gas throughout all provings, with resulting symptoms, colic pains borborygmæ; sensation of fermentation; soreness to touch on account of the pressure.

### Stool and Anus.

Noisy flatus continuous through the proving; offensive at first when the appetite was too great, and an overloaded stomach in consequence.

Early in the proving the stools were undigested and offensive; later they became hard and dark-colored; usually they would come on after eating. Whatever the stool, it would be followed by an uneasy urging as though not all was expelled. With the 40M the urging seemed at times imperative as if diarrhœa was threatening, yet nothing but flatus would be passed.

### Urine.

Sluggish stream; a few drops apt to remain and moisten the clothing; throughout the provings, often first few days. With 40M the urine caused burning or smarting in the urethra; frequent micturitions; very red for two days; saffron yellow for two days.

### Male Sexual Organs.

Sexual passion increased at first; then depressed through the proving, even to complete loss of power for many days; nocturnal emissions with the lower potencies.

### Respiratory Tract.

Hoarseness, with cough, from dryness in the trachea, which felt sore and was hurt by the cough.

Expectoration thick, white, and very scanty.

### Neck and Back.

Lymphatic glands on the back of the neck swollen (40M). Drawing pains in the lumbar region; worse by lying on the back, early in the proving; later, better by lying on the back (40M).

### Extremities.

Aching in the shoulders and arms; better by moving them; drawing aching following whole length of right sciatic nerve; better by walking slowly (30th).

Drawing pains from the knees downward, also from the soles to the knees; better by motion.

Chilliness, with severe pain from knees to ankles; could scarcely keep from groaning; after retiring, better by getting thoroughly warm (eleventh day with the 30th).

Drawing pain in the back and extremities, especially from the soles to the knees, and from the shoulders to the hands; pains frequently change location; better by warmth, also temporarily by moving affected parts. (Kept me awake from two to four A. M. seventeenth day, from three to four A. M. eighteenth day, at same time in the night, but less severe, nineteenth day, with the 40M.)

Physical prostration; the muscles feel sore if moved; better by the motion, yet quickly exhausted; feel weaker than I am in reality.

### Sleep.

Vivid, disagreeable dreams of burglars; of floods wrecking the house; of being in a public assembly unclothed.

A tendency to sleep upon the back, a position which was very uncomfortable both before and after the proving (40M).

### Fever.

Chilliness, with pains in the evening, followed by fever, sore throat, and prostration.

### Skin.

Great itching here and there; better temporarily by scratching; worse at night whenever awake (awoke with it two nights under the 40M); most severe on scalp and under the beard.

Peculiarity—an itching on the inside of the hands and feet where the skin is the thickest.

### Conditions.

Two to five A. M. aggravation of pains and itchings.

Cold air aggravates all pains.

Eating—aggravation of stomach and bowel troubles.

Aggravation of occipital headache.

Eructations of gas ameliorate stomach symptoms and headache.

Motion ameliorates pain, if gentle. Continued motion aggravates on account of muscular soreness and weakness.

Warmth ameliorates all pains.

I suggest a careful watch for a rhythmical recurrence of symptoms at an interval of from ten to fourteen days, which seems to have occurred under the influence of the 40M.

